# The complete chloroplast genome of *Achyranthes bidentata* Blume

**DOI:** 10.1080/23802359.2021.1882895

**Published:** 2021-03-12

**Authors:** Wangjun Yuan, Peng Liu, Suping Zhang, Yanxia He

**Affiliations:** a School of Pharmacy, Henan University, Kaifeng, China; b School of Life Sciences, Henan University, Kaifeng, China

**Keywords:** Chloroplast genome, *Achyranthes bidentata*, phylogenetic tree

## Abstract

*Achyranthes bidentata* is a popular Chinese medicine, that has its place in the treatment of spasm, osteodynia of the lumbar region and knees. The complete chloroplast (cp) genome of *A. bidentata* was determined. The complete cp genome is 151,543 bp in length and includes a large single-copy (LSC) region of 83,922 bp, a small single-copy (SSC) region of 17,251 bp, and a pair of inverted repeat regions (IRs) of 25,185 bp. It encodes 131 genes, including 86 protein-coding genes, eight rRNAs, and 37 tRNAs. Phylogenetic analysis indicates that four samples of *A. bidentata* formed a clade with a 100% bootstrap value.

*Achyranthes bidentata* Blume (*Amaranthaceae*), a perennial herbaceous plant, is widely distributed and grown in China (Wang et al. 2013). As a popular Chinese medicine, *A. bidentata* has its place in the treatment of spasm, osteodynia of the lumbar region and knees, and flaccidity of limbs owning to its medical functions of dissipating blood stasis, nourishing the liver and kidney, and strengthening the bones and muscles (Shen et al. 2011). It is therefore necessary to study this plant in detail. The advent of high-throughput sequencing technology makes it possible to acquire large amounts of genome data rapidly (He et al. 2017). In this study, we report the complete chloroplast (cp) genome of *A. bidentata*. The raw sequence data have been deposited into NCBI SRA with project accession of PRJNA662739, and the plastome sequence has been was registered in GenBank with the accession number MT955652.

The plant material of *A. bidentata* was sampled from the Renhe Community garden in Kaifeng city, China (34°49′8.4″N, 114°23′2.4″E), its voucher specimen (HENU20200523) has been kept at the Herbarium of Henan University. Total genomic DNA was extracted from leaf tissue with the SDS method and high-quality DNA was randomly fragmented to 350 bp. The paired-end library was then sequenced using Illumina NovaSeq PE150 at Beijing Novogene Bioinformatics Technology Co., Ltd. (Beijing, China). Approximately, 5-Gb raw data were generated, and these were trimmed and assembled into contigs using the CLC Genomics Workbench 9.5.2 (CLC Inc., Aarhus, Denmark). The complete cp genome of *A. bidentata* was later constructed using *Amaranthus caudatus* (Amaranthaceae, GenBank accession number: NC_040143) as a reference sequence and annotated using Geneious R11 (Biomatters, Auckland, New Zealand), as described by Liu et al. (2017, 2018).

**Figure 1.**
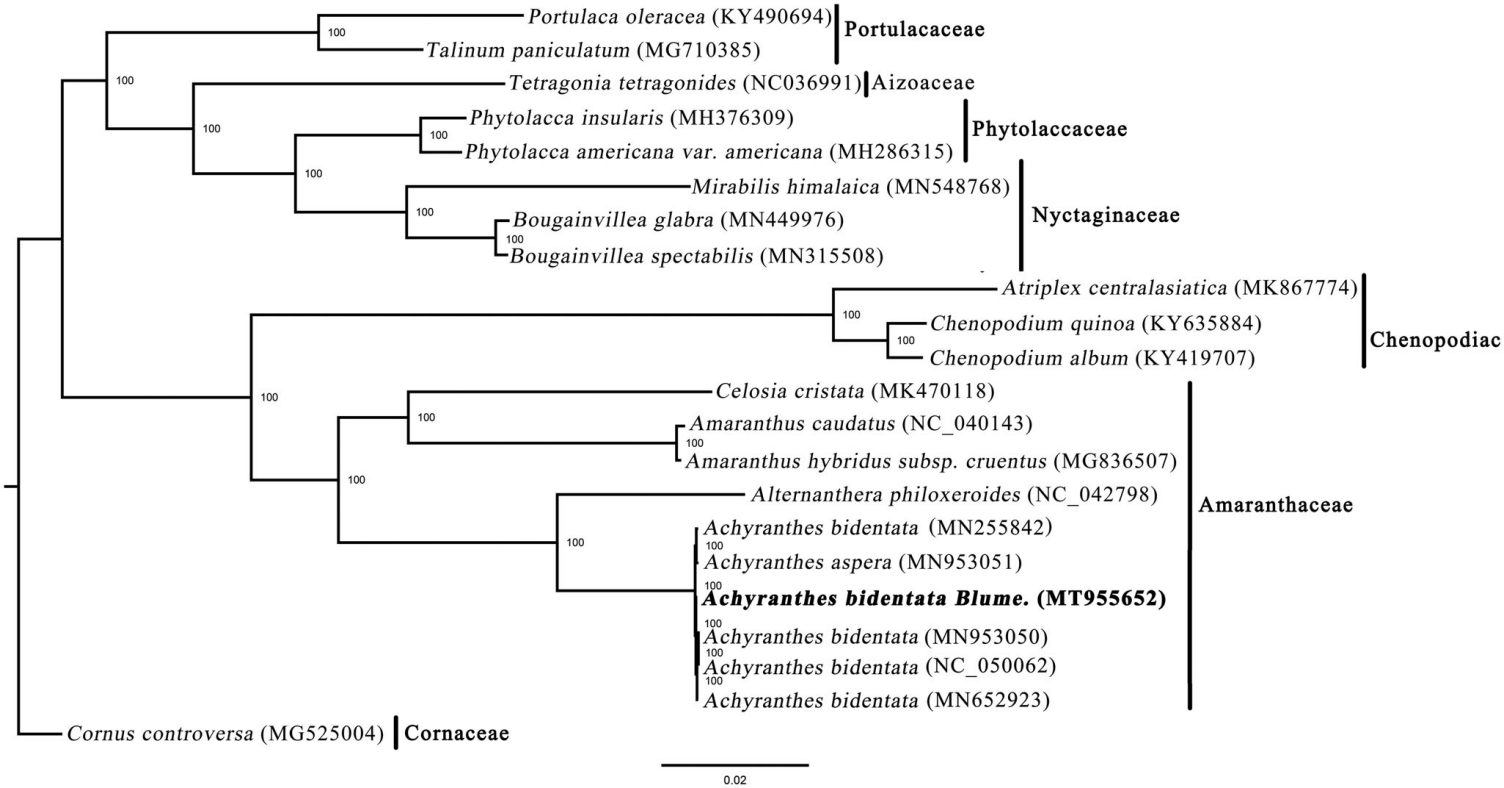
Phylogenetic relationships of Lamiales inferred based on whole chloroplast genome sequences. Number above each node indicates the ML bootstrap support values.

The complete plastome of *A. bidentata* is 151,543 bp in length and includes a large single-copy (LSC) region of 83,922 bp, a small single-copy (SSC) region of 17,251 bp, and a pair of inverted repeat regions (IRa and IRb) of 25,185 bp. The whole cp genome encoded 131 genes, including 86 protein-coding genes (PCGs), 37 tRNAs, and four rRNA operons. Among these genes, 14 genes (*atpF*, *ndhA*, *ndhB*, *petB*, *petD*, *rpoc1*, *rpl16*, *rps16*, *trnA-UGC*, *trnG-GCC*, *trnI-GAU*, *trnK-UUU*, *trnL-UAA*, and *trnV-UAC*) harbored one intron and two genes (*clpP* and *ycf3*) had two introns. Most genes occurred in a single copy; however, six PCG genes (*ndhB*, *rpl2*, *rpl23*, *rps7*, *ycf2*, and *ycf15*), seven tRNA genes (*trnA-UGC*, *trnH-CAU*, *trnI-GAU*, *trnL-CAA*, *trnN-GUU*, *trnR-ACG*, and *trnV-GAC*), and four rRNA genes (*rrn4.5*, *rrn5*, *rrn16*, and *rrn23*) in the IR regions were duplicated. The overall AT content of the cp genome is 61.9% and the corresponding values in LSC, SSC, and IR regions are 65.8, 70.0, and 57.5%, respectively. The nucleotide similarity of *A. bidentata* among the sequence we constructed and the others (MN652923, MN255842, MN953050, NC_050062) reported on NCBI is 99.8%, and there are 160 SNPS and 19 InDel in these sequences.

The phylogenetic tree, including *A. bidentata* and 16 other species in Caryophyllales, was constructed based on whole plastome sequences using RAxML-HPC XSEDE v.8.2.8 from CIPRES (http://www.phylo.org/) (Stamatakis 2014), with *Cornus controversa* as the outgroup. The tree showed four samples of *A. bidentata* formed a clade with a 100% bootstrap value (Figure 1). This result will provide valuable insight into conservation and evolutionary histories for this important species.

## Data Availability

The data that support the findings of this study are openly available in NCBI at https://www.ncbi.nlm.nih.gov. The reference numbers of the raw sequence data and the genome are PRJNA662739 and MT955652, respectively.
